# Integrated system combining cerebral protection and active steering directional puncture for thoracic aortic in situ fenestration

**DOI:** 10.1016/j.jvssci.2026.100433

**Published:** 2026-07-11

**Authors:** Xiangxiang Ru, Xinxi Li, Lei Zhang, Jingdong Tang, Shuai Jiang, Yerbao Zaiying, Gulitenken Aihemaitijiang, Donglin Li, Dilinerkezi Ablimit, Li Chen, Yuxin Deng, Halizati Halimulati, Ye Tian

**Affiliations:** a Department of Vascular and Thyroid Surgery, The First Affiliated Hospital of Xinjiang Medical University, Urumqi, Xinjiang, China; b Department of Vascular Surgery, Pudong Hospital, Fudan University, Shanghai, China

**Keywords:** Thoracic aorta, Endovascular procedures, Stent graft, In situ fenestration, Embolic protection

## Abstract

**Objective:**

This study aims to introduce and systematically evaluate the preclinical performance and safety of a novel integrated interventional device that simultaneously addresses the cerebral embolism risk and the difficulty of puncture and fenestration during thoracic aortic in situ fenestration.

**Methods:**

A study design combining in vitro experiments with anatomically accurate human aortic arch models and in vivo large animal experiments was adopted. (1) In in vitro experiments, a closed-loop pulsatile flow system was used to test the device's capture efficiency for 100 , 120 , and 150 μm embolic microspheres, and to verify its puncture accuracy for the three supra-aortic branch vessels under types Ⅰ to Ⅲ aortic arch configurations. (2) In in vivo experiments, six healthy Ukrainian White pigs were used as the animal model to assess the surgical technical success rate, procedural efficiency, operator usability feedback, and immediate safety of the device.

**Results:**

In in vitro experiments, the device achieved capture rates of (98.1 ± 0.9)% and (87.1 ± 2.7)% for the stroke-causing critical particle sizes of 150 and 120 μm, respectively, capture efficiency exhibited a size-dependent trend (*F* = 174,771; *P* < .001). Across all types Ⅰ to Ⅲ aortic arch scenarios, the puncture coverage ratio ranged from 41.9% to 68.8%, with no out-of-bounds puncture events. In in vivo experiments, the surgical technical success rate was 100%, with a mean fenestration time of only 9 seconds (range, 5-11 seconds) and a total procedural time of 38.92 ± 4.36 minutes. The overall operator usability score was 7.47 ± 0.89, among which the directional accuracy dimension received the highest score (7.83 ± 0.75). After the procedure, the device was retrieved with intact structural integrity, only mild vascular intimal injury was observed, and successful capture of thrombus debris by the embolic protection filter was confirmed.

**Conclusions:**

This integrated system offers a potential approach to the inherent challenge of incompatible access for cerebral protection and puncture during in situ fenestration procedures. Preclinical results indicate it may facilitate improved puncture success and holds potential for clinical translation.

**Clinical Relevance:**

This study addresses two core clinical challenges of thoracic endovascular aortic repair: the high risk of perioperative stroke and the technical difficulty of in situ fenestration (ISF) in complex aortic arch anatomies. The integrated device provides a novel, single-access solution that synchronizes cerebral protection and precision puncture, with the potential to improve procedural safety and efficiency, and expand the application of ISF for complex aortic pathologies.

**Article Highlights**

• **Type of Research:** Preclinical study of a novel integrated interventional device, combining in vitro bench testing and in vivo large animal experiments
• **Key Findings:** In vitro, the 120 μm mesh achieved 87.1% interception of 120 μm particles and 98.1% interception of 150 μm stroke-critical particles (particles capable of causing symptomatic cerebral ischemia); the active steering directional puncture mechanism enabled 41.9% to 68.8% puncture coverage across types Ⅰ to Ⅲ aortic arch anatomies, with no out-of-bounds puncture. In vivo, the technical success rate was 100%, with a mean fenestration time of only 9 seconds.
• **Take Home Message:** This integrated system, which synchronizes cerebral protection and active steering directional puncture, may help address the core clinical dilemma of access incompatibility between protection and puncture in conventional in situ fenestration, and provides a safe, efficient and promising technical pathway for supra-aortic branch reconstruction.

Thoracic endovascular aortic repair (TEVAR) is the first-line minimally invasive treatment strategy for thoracic aortic pathologies.^1^^,^^2^ To obtain an adequate proximal landing zone, coverage of supra-aortic branch vessels including the left subclavian artery is often required during branch reconstruction, a maneuver that predisposes patients to severe complications such as cerebral ischemia and spinal cord ischemia.^3^^,^^4^ Thus, effective intraoperative reconstruction of branch vessel blood flow is of critical importance.

In situ fenestration (ISF) has become the mainstay technique for supra-aortic branch reconstruction as it conforms to native physiological anatomy. However, it presents two key challenges: (1) Embolic risk: debris generated during stent graft puncture and aortic arch manipulation can cause cerebrovascular events, with the incidence of postoperative stroke after ISF reaching as high as 6%.^5^^,^^6^ Furthermore, there is an inherent conflict between cerebral protection and puncture fenestration during the procedure: deployment of an embolic protection filter precludes subsequent puncture, whereas performance of puncture requires abandonment of immediate cerebral protection. (2) Puncture difficulty: the complex anatomy of the aortic arch makes it difficult to align the puncture needle perpendicular to the target, which can easily lead to needle slippage, prolonged procedure time, or procedural failure. Even with dedicated devices, accurate puncture remains a major technical bottleneck.^7^, ^8^, ^9^

At present, there is no dedicated clinical device that can simultaneously resolve the aforementioned conflicts. To address this gap, we independently developed a novel integrated interventional device that combines an embolic protection filter with an active steering directional puncture catheter. Via a single vascular access, this device can simultaneously achieve “full-cycle cerebral protection during the procedure and accurate puncture under protection,” resolving the technical conflicts in conventional approaches: the incompatible access for cerebral protection and puncture, as well as the difficulty of puncture and fenestration. Through in vitro benchtop experiments and in vivo large animal experiments, this study systematically evaluated, the feasibility, technical efficiency, and preliminary safety of this integrated system during ISF for supra-aortic branch vessels, providing a potential novel technical approach for supra-aortic branch reconstruction in the thoracic aorta.

## Methods

This study adopted a two-stage preclinical study design consisting of in vitro performance validation and in vivo procedural and safety validation, to systematically evaluate the effectiveness, procedural feasibility, and immediate safety profile of the integrated cerebral protection and active steering directional puncture system. All experimental procedures were strictly performed in accordance with the international guidelines for preclinical development of interventional devices and the ethical requirements for laboratory animal welfare.

### In vitro experiments

#### Experimental materials and equipment

Core devices: integrated cerebral protection and active steering directional puncture system for thoracic aortic ISF (Suzhou Dingke Medical Technology Co, Ltd). The core innovation of this system is the simultaneous realization of full-cycle intraoperative embolic protection and directional puncture fenestration via a single vascular access. Its core functional units include a 120 μm-pore embolic protection filter, an active steering directional puncture assembly, and matched delivery and retrieval assemblies. A commercial thoracic aortic stent graft (Medtronic) was used as the supporting device for in vitro experiments.

##### Experimental materials

Fluorescent polystyrene microspheres (diameters: 100 , 120 , and 150 μm, Beijing Zhongke Keyou Technology Co, Ltd); glycerol, deionized water, Tween-20 [Youtepu Technology (Suzhou) Co, Ltd]; blood analog solution (glycerol:deionized water = 4:6).

##### Key equipment

Self-built closed-loop pulsatile flow simulation system (pulsation frequency, 60 beats/min; blood flow velocity, 50-150 mL/s; pressure range, 80-120 mmHg); transparent polymer anatomically accurate human aortic arch model (reproducing the anatomical structures and branch angles of the aortic arch, brachiocephalic trunk, left common carotid artery, and left subclavian artery, including types Ⅰ-Ⅲ aortic arch configurations), fabricated based on computed tomography angiographic data from patients with representative aortic arch anatomies; particle size analyzer (Tianjin Tianhe Analytical Instrument Co, Ltd); ImageJ version 1.8.0 image analysis software (National Institutes of Health).

#### Simulation system and clinical scenario model construction

The anatomically accurate human aortic arch model was hermetically connected to the circuit of the closed-loop pulsatile flow system, followed by perfusion with the blood analog solution and complete removal of air from the circuit. The pulsatile pump was then activated and calibrated to human physiological hemodynamic parameters (pulsation frequency, 60 beats/min; mean flow velocity, 80 mL/s; mean arterial pressure, 100 mmHg), with a stable circulatory environment maintained for 30 minutes. The commercial thoracic aortic stent graft was accurately implanted into the aortic arch model in strict accordance with the manufacturer's instructions for use, to simulate the standard clinical surgical scenario of stent coverage of the supra-aortic branch ostia after thoracic endovascular aortic repair.

#### Quantitative analysis of embolus capture efficiency

A randomized controlled design was adopted, with an experimental group (filter deployed) and a blank control group (no filter deployed). For each microsphere size in each group, three independent replicate experiments were performed (n = 3), and the experimental sequence was randomized using a computer-generated random sequence to avoid systematic error.

##### Experimental pretreatment

Fluorescent microsphere suspensions of 100 , 120 , and 150 μm were prepared at a concentration of 100 particles/mL, followed by sonication to ensure uniform dispersion of the microspheres without sedimentation. The closed-loop flow system and all catheter accesses were rinsed 3 times with deionized water, then preperfused with the blood analog solution to maintain stable hemodynamic parameters. All sample collection tubes were rinsed with deionized water containing 0.1% Tween-20 and air-dried to reduce microsphere adsorption to the tube wall.

##### Experimental procedure

The integrated system was introduced into the aortic arch model via the simulated brachial artery access. The embolic protection filter was then precisely positioned 5 mm proximal to the origin of the vertebral artery within the left subclavian artery; full deployment, good wall apposition, and stable positioning were confirmed. A 5F single-curve catheter was placed via the simulated femoral artery access, with the catheter tip fixed 1 cm proximal to the filter. After uniform injection of 1 mL of microsphere suspension, 2.5 mL of deionized water was injected at the same flow rate to flush residual microspheres from the circuit. At 2 seconds after microsphere injection (time determined by pre-experiments), the total effluent was hermetically collected at the interface of the vertebral artery outflow tract into a 10 mL graduated tube.

##### Data quantification

A particle size analyzer was used to count the microspheres in all collected effluent samples, and the capture efficiency was calculated using the following formula: Capture efficiency (%) = [1 – {(Mean number of microspheres in the effluent of the experimental group)/(Mean number of microspheres in the effluent of the control group)} × 100%].

#### Performance evaluation of active steering directional puncture

Model preparation: In the aortic arch model with an implanted thoracic aortic stent graft, the stent graft regions corresponding to the ostia of the left subclavian artery, left common carotid artery, and brachiocephalic trunk were localized respectively to simulate types Ⅰ to Ⅲ aortic arch anatomical configurations.

##### Experimental procedure

(1) The integrated system was delivered to the stent graft site corresponding to the target branch vessel, with the tip of the active steering directional puncture catheter positioned against the graft membrane; (2) the force direction and angle of the catheter tip were adjusted via the matched control system to complete simulated puncture; (3) the spatial distribution of all successful puncture sites was recorded.

##### Area calculation

(1) Accessible puncture area: a convex polygon was formed by connecting all successful puncture sites, and the area was calculated using ImageJ software; (2) target projection area: the geometric projection area of the target vessel ostium on the stent graft membrane was measured; (3) effective puncture area ratio = [(Accessible puncture area)/Target projection area) × 100%].

The effective puncture area ratio is an engineering metric that quantifies how concentrated the puncture attempts are within the projected ostium of the target vessel. A higher ratio suggests that the active steering mechanism can help direct the puncture needle toward the intended target zone. This capability could potentially reduce the likelihood of off-target puncture—a challenge frequently encountered during ISF in complex arch anatomies. However, the clinical relevance of this metric remains to be established.

#### Qualitative observation of air embolus capture

The embolic protection filter was stably deployed and fully expanded in a straight-tube flow circuit system, with the simulated physiological blood flow environment maintained. 0.1 mL of air was rapidly injected into the flow circuit proximal to the filter to generate air bubbles. The dynamic interaction between the air bubbles and the filter mesh was observed under direct visualization, and the final state of the air bubbles (ie, entrapment, fragmentation, and distal escape) was qualitatively recorded.

### In vivo experiments

#### Experimental animals and ethical approval

All experimental protocols were approved by the Laboratory Animal Ethics Committee of Shanghai Key Laboratory of Vascular Surgery (Approval No. 2025-MS-LAT-D-62). All procedures were performed in strict accordance with the 3R Principles for the Welfare and Use of Laboratory Animals, and the Guide for the Care and Use of Laboratory Animals (Institute of Laboratory Animal Resources, National Research Council, 1996).

#### Rationale for animal model selection and study design

In this study, an abdominal aortoiliac artery bifurcation model was used to simulate the procedural workflow of thoracic aortic ISF, with the core rationale as follows: (1) the angle between the branch vessel and the aorta in this model is highly consistent with that of the human type Ⅲ aortic arch, which can fully replicate the entire procedural workflow of clinical ISF, including “stent graft coverage of the branch vessel ostium-embolic protection filter deployment-directional puncture-balloon dilation-branch stent implantation”; (2) this model can eliminate the interference of the complex curved anatomy of the aortic arch on the procedural learning curve, enabling more objective and accurate evaluation of the procedural feasibility, efficiency, and immediate safety profile of the device itself; (3) in this study, core performance validation of the device in real clinical anatomical scenarios has been completed via the in vitro anatomically accurate human types Ⅰ to Ⅲ aortic arch models. The core objective of the in vivo experiments is to verify the procedural feasibility and biosafety of the device in a living environment, and this model fully aligns with the objectives of the study design.

#### Surgical instruments and experimental reagents

##### Surgical instruments

Abdominal aortic bifurcation stent graft system with iliac limbs (Medtronic); hydrophilic super-stiff guidewires (Terumo), Anplus stiff guidewires (Terumo), hydrophilic guidewires × 6 (Terumo); 5F angiographic catheters × 6 (Johnson & Johnson), 5F single-curve catheters × 6 (Johnson & Johnson); 2 mm Sterling balloon catheters × 6 (Boston Scientific); 4 mm peripheral balloon dilatation catheters × 6 (Dingke); 7 mm peripheral balloon dilatation catheters × 6 (Dingke); indeflators × 6 (Dimark); self-expanding bare metal stents × 6 (Bard).

##### Experimental reagents

Zoletil 50, for induction of anesthesia (Virbac); Xylazine hydrochloride, for induction of anesthesia (Shengda Animal Pharmaceutical Co, Ltd); Isoflurane, for maintenance of anesthesia (RWD Life Science Co, Ltd); Heparin sodium injection, for anticoagulation (Hepalink Pharmaceutical Group Co, Ltd); Iopamidol contrast medium (Bracco Sine Pharmaceutical Co, Ltd); Lidocaine, for vasodilation (Hualu Pharmaceutical Co, Ltd); Atropine (Changjiang Pharmaceutical Co, Ltd); 0.9% Normal Saline, for fluid resuscitation (Kaile Biotechnology Co, Ltd); 10% Neutral Buffered Formalin, for tissue fixation (Solarbio Science & Technology Co, Ltd); Hematoxylin and Eosin (H&E) Staining Kit (Absin Bioscience (Shanghai) Co, Ltd); Elastic Fiber Staining Kit (Solarbio Science & Technology Co, Ltd).

#### Surgical procedure

**Fig 1:**
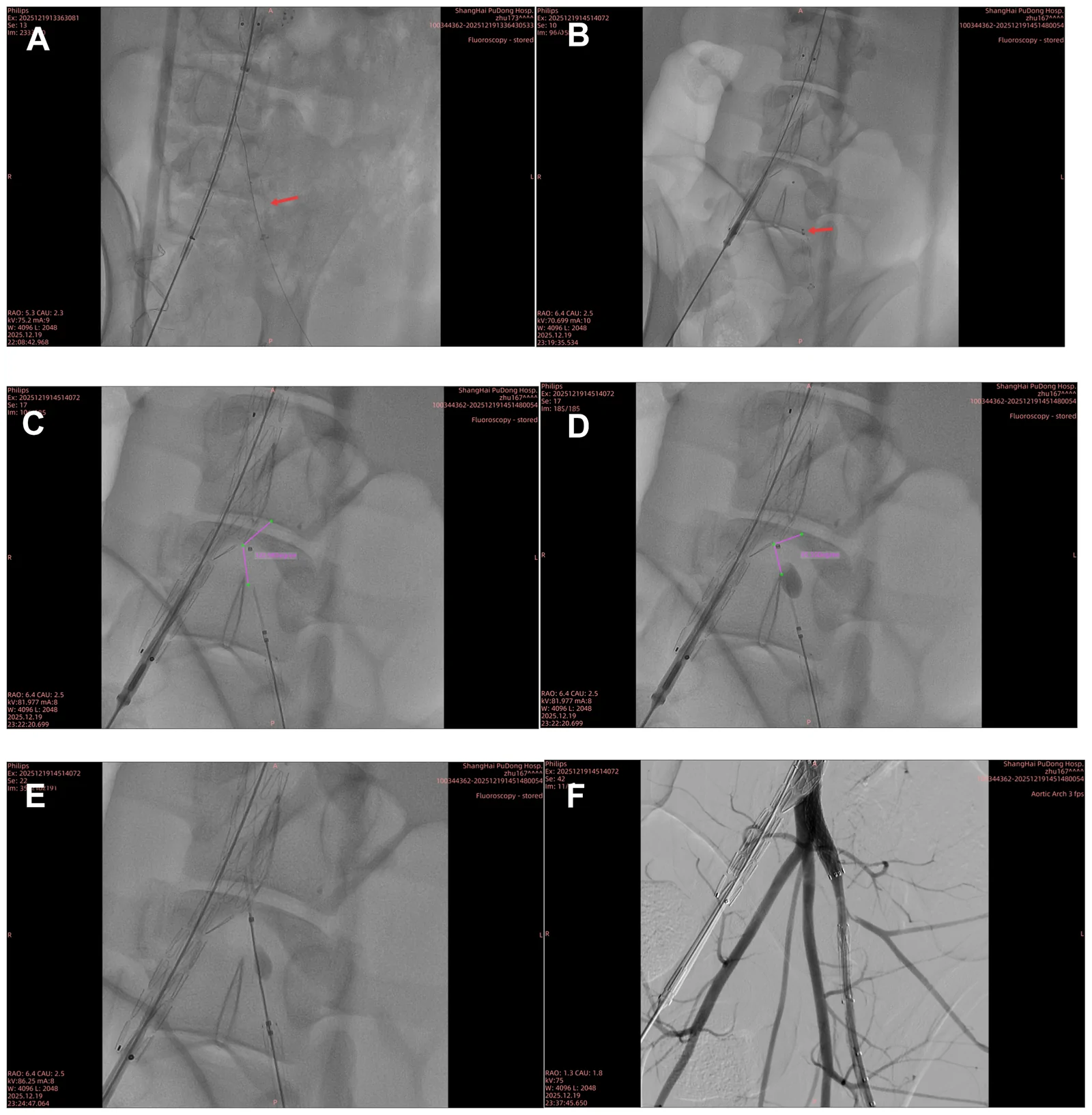
Digital subtraction angiography (DSA) images of key procedural nodes of the integrated system during in vivo operation. **A,** Angiography after precise deployment of the embolic protection filter, confirming favorable deployment morphology and accurate positioning of the filter. The *red arrow* marks the position of the embolic protection filter; **B,** Angiography after the active steering catheter was delivered to the target position through the central lumen of the embolic protection filter. The *red arrow* indicates the position of the steerable catheter; **C,** Before steering adjustment, the catheter tip presented an obtuse angle with the tangent line of the stent graft; **D,** After directional steering adjustment, the catheter tip was perpendicular to the tangent line of the stent graft, creating conditions for successful puncture and fenestration; **E,** Successful puncture and membrane fenestration, with the guidewire smoothly passing through the fenestration and entering the target vessel; **F,** Final angiography after branch stent deployment, confirming unobstructed blood flow in the target iliac artery without endoleak or stenosis.

All surgeries were performed by two senior attending vascular surgeons, with the entire procedure monitored under a digital subtraction angiography system. The core workflow is as follows (angiography of key procedural nodes is shown in Fig 1).

##### Anesthesia and preparation

Intramuscular injection of Zoletil 50 (0.75 mg/kg) combined with xylazine hydrochloride (6 mg/kg) was administered for anesthesia induction. After entering the operating room, the animal underwent endotracheal intubation, and anesthesia was maintained with 1% to 3% inhaled isoflurane. The bilateral inguinal regions were prepped and sterilized; the skin was incised to dissect the external iliac arteries, which were suspended with rubber bands for subsequent use.

##### Access establishment

Surgical puncture of the suspended external iliac artery was performed under direct vision, and a 6F sheath was inserted to establish the surgical access.

##### Main stent implantation

An angiographic catheter was advanced via the left access to perform abdominal aortography; the vessel diameter was measured, and a size-matched abdominal aortic stent was selected. The catheter was exchanged for an Anplus stiff guidewire, and the stent was advanced into the abdominal aorta with deployment deferred temporarily.

##### Integrated system deployment

The integrated system was delivered to the target iliac artery via the right femoral artery access. The filter delivery catheter was fixed, the outer sheath was retracted, and the embolic protection filter was precisely deployed approximately 3 cm proximal to the target vessel ostium.

##### Directional puncture and fenestration

(1) The puncture adjustment catheter was advanced to the corresponding site of the stent graft membrane via the central lumen of the filter. The stent was deployed along the stiff guidewire, with the main body of the stent positioned in the abdominal aorta, the unilateral iliac limb extended into the ipsilateral iliac artery, and the ostium of the contralateral iliac artery retained as the simulated “target branch vessel.” (2) The tip angle of the active steering directional puncture catheter was adjusted to position it as perpendicular to the graft membrane as possible. (3) Timing of fenestration was initiated, the graft membrane was punctured with a puncture needle, timing was stopped, and a 0.018-inch guidewire was advanced through the fenestration. (4) The puncture needle and active steering directional puncture catheter were withdrawn.

##### Branch vessel reconstruction

Sequential dilation of the puncture site was performed with 4 and 7 mm peripheral balloons in turn. A self-expanding bare metal stent was advanced over the guidewire and deployed at the fenestration site to complete branch vessel reconstruction.

#### Endpoint assessment and data collection

##### Feasibility endpoint

Technical success rate was calculated with the formula below: Technical success rate (%) = [(Number of experimental animals that successfully completed the full workflow of “embolic protection filter deployment-directional puncture-branch stent implantation”)/(Total number of experimental animals) × 100%].

##### Efficiency endpoints

(1) Puncture and fenestration time: the total time from the completion of adjustment of the active steering directional puncture catheter to the passage of the guidewire through the graft membrane was recorded. (2) Operator usability feedback scale was designed with reference to the subjective operation scoring scale for other developed devices^10^ (as shown in Table I). To collect preliminary user experience, the lead surgeon scored the operational experience of the device postoperatively using a 0-10 point visual analog scale (0 points = unable to operate, 10 points = extremely easy to operate), with assessments across five dimensions: overall usability, directional accuracy, procedural simplification, operability of the cerebral protection device, and confidence in safety.

**Table I:** Operator usability feedback rating scale

Assessment dimension	Core question	Scoring reference	Score
Overall usability	Is the overall operation process of the entire device from delivery to retrieval intuitive and smooth?	0-3 points: Chaotic process with extreme operational difficulty	
		4-6 points: Feasible process but requiring extensive adjustments	
		7-8 points: Smooth process, basically meeting expectations	
		9-10 points: Highly intuitive and efficient process	
Operability of the cerebral protection device	Is the deployment, positioning, and retrieval process of the embolic protection filter controllable and reliable?	0-3 points: Uncontrollable, with failed positioning or difficult retrieval	
		4-6 points: Controllable but requiring repeated adjustments, with low confidence in reliability	
		7-8 points: Controllable and reliable, with successful completion on the first attempt	
		9-10 points: Precise and smooth operation with excellent handling experience	
Directional accuracy of the active steering directional puncture catheter	Is the function of adjusting the catheter tip angle and puncture force line via inflation of different balloons accurate and effective?	0-3 points: Unable to achieve effective orientation, or extremely unstable	
		4-6 points: Able to achieve orientation but with slow response or moderate accuracy	
		7-8 points: Able to effectively and stably achieve a vertical puncture force line	
		9-10 points: Rapid response, high precision, and excellent handling performance	
Degree of procedural simplification	Does this integrated device truly simplify the coordinated operation of “cerebral protection” and “precise puncture”?	0-3 points: No simplification achieved, even more complicated than conventional procedures	
		4-6 points: Mild simplification with no obvious advantage	
		7-8 points: Significantly simplified workflow and reduced operational burden	
		9-10 points: Revolutionary simplification that renders complex steps straightforward	
Confidence in safety	How confident are you in the safety of the device throughout the entire operation?	0-3 points: Concerns about safety with clear potential risks	
		4-6 points: Generally safe but with residual concerns	
		7-8 points: Confident in safety, with comprehensive safety considerations in the device design	
		9-10 points: Extremely high confidence in safety with a strong sense of security	

##### Safety endpoints

(1) Device integrity: immediately after the procedure, the retrieved active steering directional puncture catheter and embolic protection filter were macroscopically examined, and the presence or absence of coating exfoliation and structural damage was recorded and photographed. (2) Embolus capture analysis: the embolic protection filter was gently flushed, and the flushing fluid was collected, observed and photographed under a microscope, with the presence or absence of captured debris recorded. (3) Histopathological evaluation: immediately after the procedure, the iliac artery segment containing the puncture site was harvested, fixed in 10% neutral buffered formalin, embedded in paraffin, and sectioned, followed by H&E staining and elastic fiber staining. The integrity of the vascular intima, elastic lamina rupture, and the degree of acute inflammatory reaction were evaluated under a microscope.

### Statistical analysis

Statistical analysis was performed using SPSS version 26.0 software (IBM Corp.). Continuous variables conforming to normal distribution were expressed as means ± standard deviation, data with non-normal distribution were expressed as median (interquartile range), and categorical variables were expressed as number (%). For the comparison of capture efficiency of microspheres with different particle sizes, the normality of the data was first verified by the Shapiro-Wilk test, and the homogeneity of variances was verified by Levene test. One-way analysis of variance was applied for data conforming to normal distribution with homogeneous variances, with the Bonferroni method for pairwise comparisons. For data that did not meet the above criteria, the Kruskal-Wallis *H* test was used, with the Dunn method for pairwise comparisons. Spearman rank correlation analysis was performed to evaluate the correlation between microsphere particle size and capture efficiency. A two-sided *P* < .05 was considered statistically significant. However, given the small sample size, all statistical analyses are exploratory and results should be interpreted as descriptive rather than definitive.

## Results

In this study, a two-stage study design consisting of in vitro experiments with anatomically accurate human aortic arch models and in vivo large animal experiments was adopted to systematically evaluate the core preclinical performance of the integrated system, and all experiments were successfully completed. In vitro experiments verified the embolic capture efficacy of the device and its puncture accuracy under types Ⅰ to Ⅲ aortic arch configurations. During in vivo experiments, the animals maintained stable vital signs with no intraoperative adverse events, and the surgical technical success rate was 100%.

### In vitro experimental results

#### Embolus capture efficiency

A paired design was used to evaluate the capture rate for each microsphere size. Capture rate was calculated as: [(Blank control count − experimental count)/(Blank control count) × 100%]. The raw data of each particle size group and the descriptive statistical results of the capture rate are shown in Table II. As the particle size of the fluorescent microspheres increased from 100 to 150 μm, the capture rate showed a significant gradient increase: the mean value was (24.2 ± 8.2)% in the 100 μm group (range, 16.3%-33.3%), (87.1 ± 2.7)% in the 120 μm group (range, 84.2%-89.7%), and reached (98.1 ± 0.9)% in the 150 μm group (range, 97.2%-99.0%). The three groups of data showed small intragroup fluctuation with no outliers.

**Table II:** Descriptive statistical results of capture efficiency

Microsphere particle size (μm)	No. of microspheres in the blank control group (counts)	No. of microspheres in the experimental group (counts)	Capture efficiency (%)
100	96.3 ± 9.6	72.7 ± 8.0	24.2 ± 8.2
120	100.3 ± 3.1	13.3 ± 2.5	87.1 ± 2.7
150	99.7 ± 4.0	2.0 ± 1.0	98.1 ± 0.9

#### Normality and homogeneity of variance test

The Shapiro-Wilk test showed that the capture efficiency of the three groups all conformed to normal distribution (100 μm: *W* = 0.999, *P* = .942; 120 μm: *W* = 0.893, *P* = .363; 150 μm: *W* = 0.964, *P* = .637; all *P* > .05). Homogeneity of variances was verified by Levene test, and the results showed homogeneous variances among the three groups (*F* = 3.555; *P* = .092 > .05), which met the prerequisites for parametric test.

#### Validation of intragroup capture effect (blank control group vs experimental group)

Paired *t*-test results showed that the capture effect was notable in the 120 μm group (*t* = 56.955; *d*_*f*_ = 2; *P* < .001) and the 150 μm group (*t* = 55.372; *d*_*f*_ = 2; *P* < .001); a certain capture effect was also observed in the 100 μm group (*t* = 4.313; *d*_*f*_ = 2; *P* = .050; Table III).

**Table III:** Results of paired *t*-test for capture efficiency

Microsphere particle size (μm)	*t* value	Degrees of freedom (*d*_*f*_)	*P* value	95% CI
100	4.313	2	.05	−0.012-9.512
120	56.955	2	<.001	83.025-91.175
150	55.372	2	<.001	96.834-99.366

*CI*, Confidence interval.

#### Comparison of intergroup differences in capture efficiency

**Fig 2:**
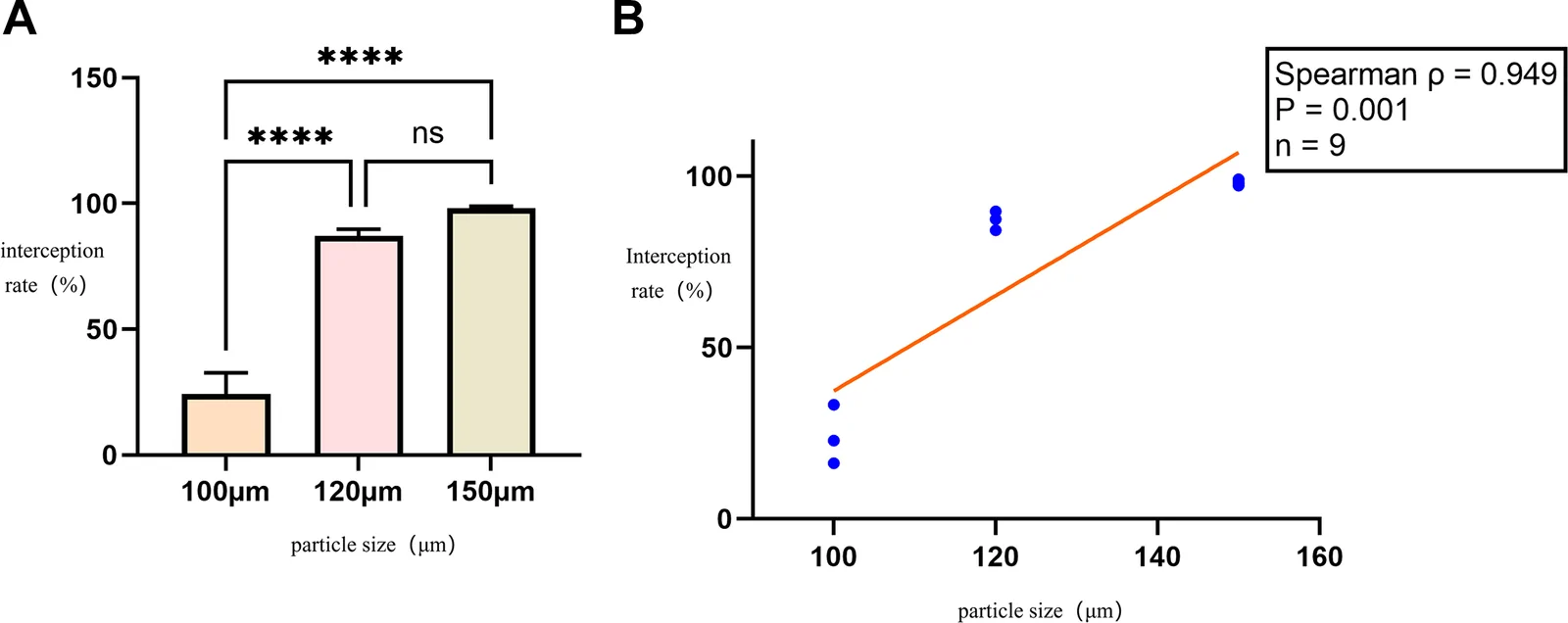
Capture efficiency of the integrated system for embolic microspheres of different particle sizes and correlation analysis. **A,** Intergroup comparison of capture efficiency for microspheres of different particle sizes. The differences in capture rate between the 120 and 150 μm microsphere groups and the 100 μm microsphere group were extremely statistically significant (∗∗∗∗*P* < .001); **B,** Spearman rank correlation analysis between the particle size of fluorescent microspheres and capture efficiency, which showed a strong positive correlation between the two (*r*_*s*_ = 0.949, n = 9; *P* < .001). The experiment was performed using an embolic protection filter with a 120 μm pore size, with three independent repeated experiments conducted for each particle size in each group.

One-way analysis of variance showed that capture efficiency differed among the three groups (*F* = 174.771; *d*_*f*_ = 2; 6, *P* < .001), with rates increasing as particle size increased. The results of post hoc multiple comparisons using the Tukey honestly significant difference method were as follows: the differences between the 100 μm group and 120 μm group (*P* < .001), as well as between the 100 μm group and 150 μm group (*P* < .001), were both extremely statistically significant. There was no statistically significant difference in capture efficiency between the 120 μm group and 150 μm group (Tukey honestly significant difference method, *P* = .093); only the uncorrected least significant difference method for multiple testing indicated a statistically significant intergroup difference (*P* = .042). The overall results still confirmed the dose-effect trend that capture efficiency increased with the increase of microsphere particle size (Fig 2, *A*).

#### Correlation between particle size and capture efficiency

Spearman rank correlation analysis showed that there was a strong positive correlation between the particle size of fluorescent microspheres and capture efficiency (*r*_s_ = 0.949; n = 9; *P* < .001), indicating that particle size was a key factor influencing the filter capture efficacy (Fig 2, *B*).

#### Performance of active steering directional puncture

**Fig 3:**
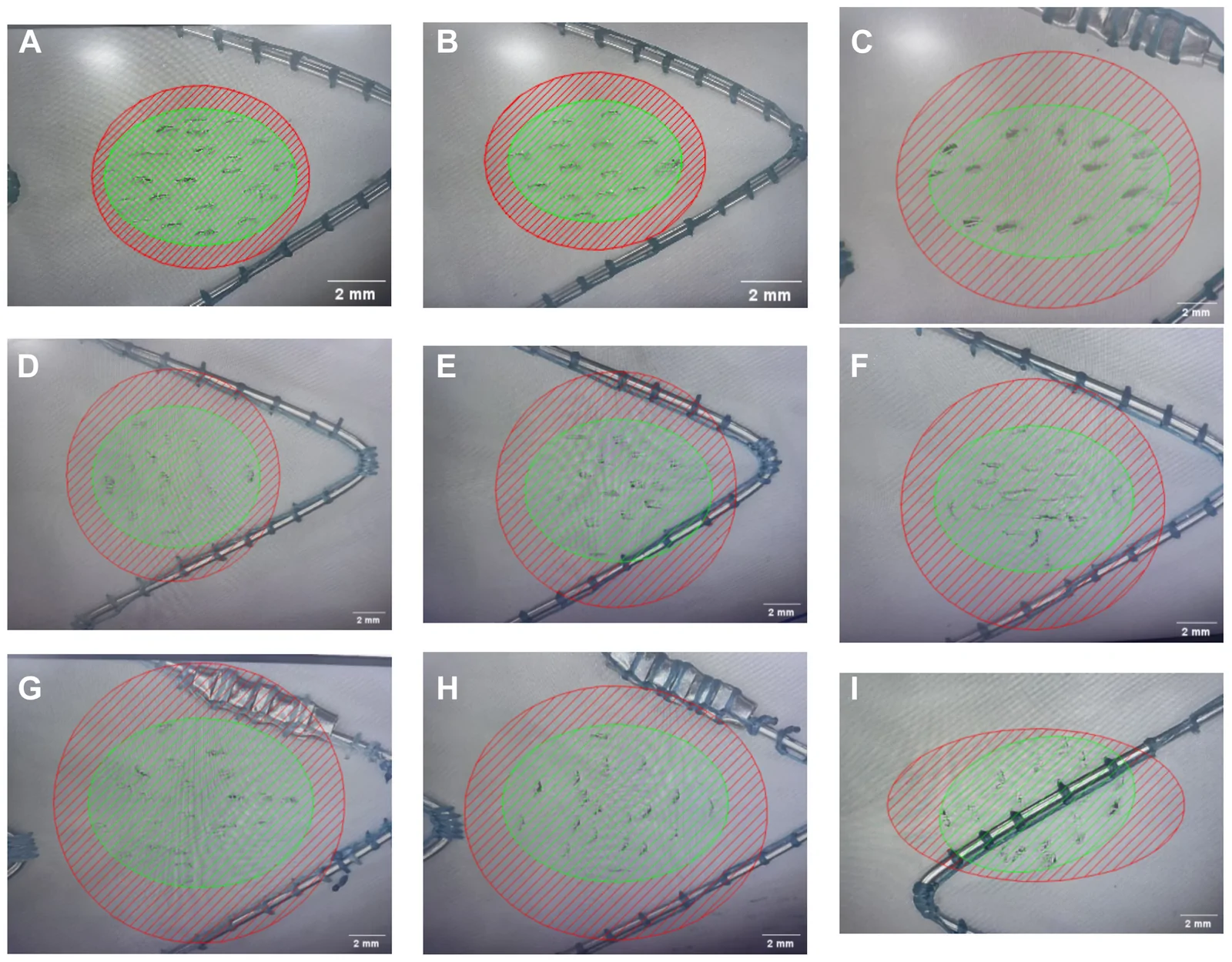
Spatial distribution of puncture sites for the three supra-aortic branch vessels under types Ⅰto Ⅲ aortic arch anatomies, demonstrating the directional accuracy and concentration performance of the active steering puncture mechanism. **A-C,** Puncture coverage areas of the left subclavian artery under types Ⅰto Ⅲ aortic arches, respectively; **D-F,** Puncture coverage areas of the left common carotid artery under types Ⅰto Ⅲ aortic arches, respectively; **G-I,** Puncture coverage areas of the brachiocephalic trunk under types Ⅰto Ⅲ aortic arches, respectively. *Red* hatched areas represent the geometric projection of the target vessel ostium on the stent graft membrane; *green* solid areas represent the actual distribution range of all successful puncture sites. Across all nine test scenarios, all puncture sites were concentrated within the projection area of the target vessel ostium with no out-of-bounds puncture events.

The in vitro experiments systematically evaluated the puncture concentration performance of this integrated system for the three main supra-aortic arteries (such as left subclavian artery, left common carotid artery, and brachiocephalic trunk) under three aortic arch configurations (types I-Ⅲ). The results of the core measurement indicator “puncture coverage ratio” (area of the puncture range/projection area of the artery ostium) are shown in Fig 3 and Table IV.

**Table IV:** Puncture performance parameters of the integrated system for supra-aortic arteries under different aortic arch configurations

Artery type	Aortic arch types	Projection area of artery ostium, mm^2^	Area of puncture range, mm^2^	Puncture coverage ratio, %
Left subclavian artery	Ⅰ	70.69	48.63	68.8
	Ⅱ	72.81	38.64	53.1
	Ⅲ	74.93	31.42	41.9
Left common carotid artery	Ⅰ	63.62	33.34	52.4
	Ⅱ	62.49	29.32	46.9
	Ⅲ	60.58	26.68	44.0
Brachiocephalic trunk	Ⅰ	91.84	42.85	46.7
	Ⅱ	79.74	36.81	46.2
	Ⅲ	48.99	28.01	57.2

Across all nine test scenarios, the measured values of the puncture coverage ratio ranged from 41.9% to 68.8% (Table IV), indicating that all puncture sites were highly concentrated within the projection area of the target vessel ostium, with no out-of-bounds puncture observed. With the increase in the anatomical complexity of the aortic arch, the puncture concentration of different arteries presented a differentiated trend.

For the left subclavian artery and left common carotid artery, the measured values of the puncture coverage ratio showed a gradual decreasing trend as the arch configuration evolved from type Ⅰ to type Ⅲ. The ratio for the left subclavian artery decreased from 68.8% in the type Ⅰ arch to 41.9% in the type Ⅲ arch, whereas the ratio for the left common carotid artery decreased from 52.4% to 44.0%.

**Fig 4:**
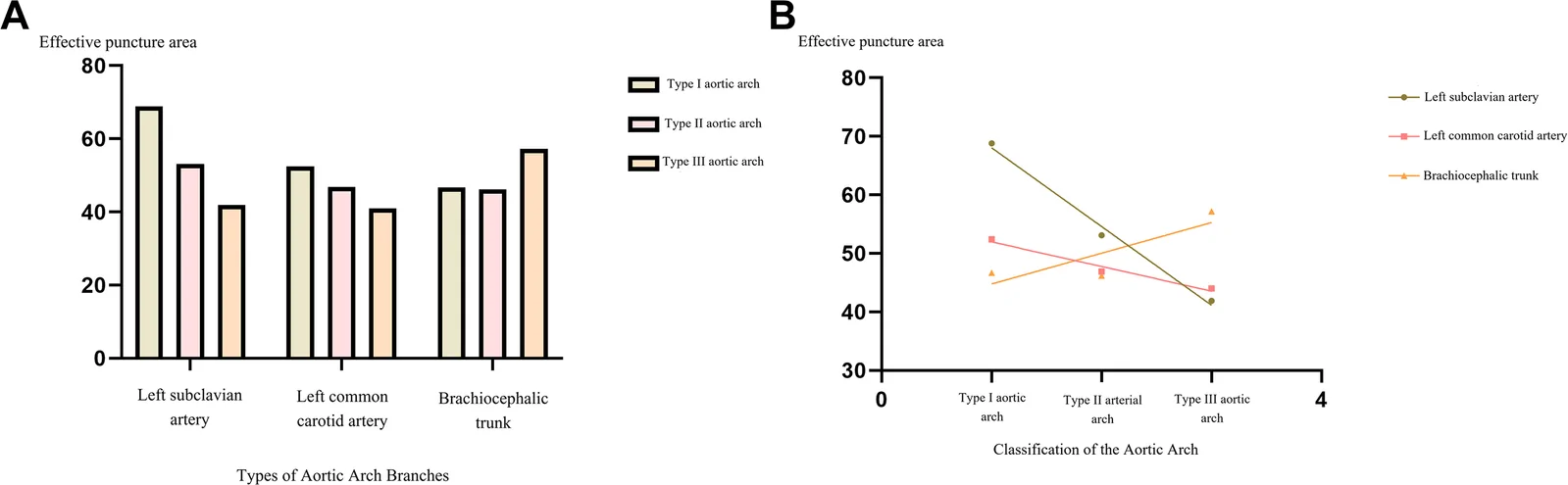
Changing trend of effective puncture coverage ratio of supra-aortic branch vessels under different aortic arch configurations. **A,** Bar graph of the puncture coverage ratio of different branch vessels under types Ⅰ to Ⅲ aortic arches; **B,** Trend line graph of the puncture coverage ratio changing with the anatomical complexity of the aortic arch. The left subclavian artery and left common carotid artery showed a monotonic decreasing trend, whereas the brachiocephalic trunk presented a “V-shaped” trend of initial decrease followed by rebound. The X-axis represents the aortic arch type, and the Y-axis represents the puncture coverage ratio (%).

Of particular note were the results for the brachiocephalic trunk, which presented a variation pattern distinct from the two aforementioned arteries: the puncture coverage ratio reached the minimum value (46.2%) under the type Ⅱ arch, whereas under the type Ⅲ arch with the most restricted anatomical space, this ratio increased to 57.2% (Fig 4, *A*).

##### Visualization analysis of puncture concentration trend

To intuitively demonstrate the variation trend of the above measured values with the anatomical complexity of the aortic arch, a scatter and line plot of the puncture coverage ratio was generated with arch type (types Ⅰ-Ⅲ) set as the ordinal variable (Fig 4, *B*).

As shown in Fig 4, the connecting line of the datapoints for the left subclavian artery and left common carotid artery presented a clear negative monotonic trend. In contrast, the connecting line of the data points for the brachiocephalic trunk clearly showed a non-monotonic “V-shaped” pattern: a slight decrease from type Ⅰ to type Ⅱ arch, followed by a marked rebound in the type Ⅲ arch. This visualization result corroborated the aforementioned findings, indicating that under the extreme condition of significant compression of the aortic arch anatomical space (the projection area of the brachiocephalic trunk in the type Ⅲ arch was reduced by approximately 46.7% compared with that in the type Ⅰ arch), the variation pattern of puncture concentration for the brachiocephalic trunk was different from that of the left subclavian artery and left common carotid artery.

#### Qualitative observation of air embolus capture

This part was a preliminary experiment designed to preliminarily observe the potential capture effect of the embolic protection filter on air embolic. The fully deployed embolic protection filter achieved fragmentation and partial entrapment of injected air bubbles; however, the clinical relevance of this observation requires further investigation in larger, quantitative studies.

### In vivo experimental results

#### General conditions and feasibility endpoint

All six female Ukrainian Large White pigs enrolled in this study successfully completed the full simulated ISF and vascular reconstruction workflow of “embolic protection filter deployment-directional puncture-branch stent implantation,” with no operation failure, device-related complications, or experimental interruption events. The technical success rate in the model reached 100%, which verified the feasibility of the basic operation of the device.

#### Efficiency endpoint

The statistical results of the core operational efficiency indicators are shown in Table V.

**Table V:** Statistical results of core operational efficiency indicators

Observation indicator	Mean ± standard deviation	Range
Time from adjustment completion to puncture and fenestration, minutes	0.15 ± 0.03	0.08-0.18
Total operative time, minutes	38.92 ± 4.36	34.90-45.72

**Fig 5:**
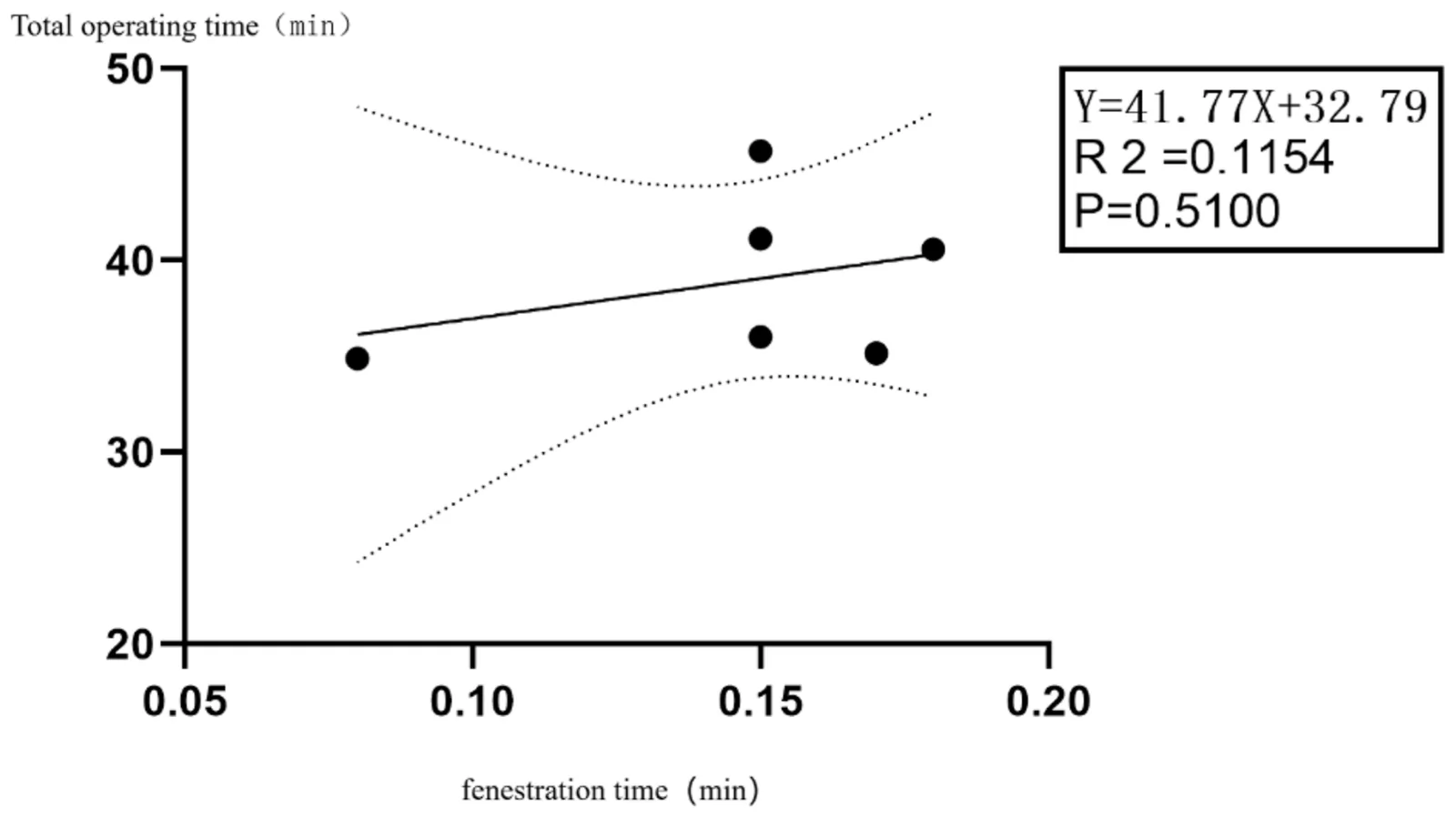
Linear correlation analysis between puncture and fenestration time and total operative time. The X-axis represents puncture and fenestration time (minutes), and the Y-axis represents total operative time (minutes); the regression equation is Y = 41.77X + 32.79, with a coefficient of determination *R*^*2*^ = 0.1154 and *P* = .51, indicating no significant linear correlation between the two (n = 6).

Time from adjustment completion to puncture and fenestration: The mean value was 0.15 ± 0.03 minutes (range, 0.08-0.18 minutes, ie, 5-11 s), indicating stable and highly time-efficient performance of this key step. Total operative time: the mean value was 38.92 ± 4.36 minutes (range, 34.90-45.72 minutes), with controllable duration of the overall workflow. Linear correlation analysis based on the six experimental animals in this study showed no significant linear correlation between fenestration time and total operative time (*R*^2^ = 0.1154; *P* = .51), indicating that under the experimental conditions of this study, the duration of this step had no significant impact on the overall operative duration (Fig 5).

#### Operator usability feedback rating

As an exploratory assessment to gather preliminary feedback on device design, two attending surgeons rated multiple dimensions of device usability on a 0 to 10-point visual analog scale (0 = unable to operate; 10 = extremely easy to operate) after each procedure. These ratings represent preliminary observational data only and should not be interpreted as a definitive usability validation.

As shown in Table VI, the mean score of all dimensions ranged from 7.17 to 7.83 points. Among them, the dimension of “Directional Accuracy of the Active Steering Directional Puncture Catheter” received the highest score (7.83 ± 0.75 points), followed by “Overall Usability” and “Degree of Procedural Simplification” (both 7.50 points). Verbal feedback from the operators indicated that the integrated design reduced the steps of device exchange, and the active steering function helped to establish a stable puncture angle and boost operational confidence before puncture.

**Table VI:** Descriptive statistical table of operator usability feedback (mean ± standard deviation)

Rating dimension	Surgeon 1 (mean ± standard deviation)	Surgeon 2 (mean ± standard deviation)	Total (mean ± standard deviation)
Overall usability	6.67 ± 0.58	8.33 ± 0.58	7.50 ± 1.05
Operability of the cerebral protection device	6.33 ± 1.15	8.00 ± 1.00	7.17 ± 1.33
Directional accuracy of the active steering directional puncture catheter	7.33 ± 0.58	8.33 ± 0.58	7.83 ± 0.75
Degree of procedural simplification	7.00 ± 0.00	8.00 ± 0.00	7.50 ± 0.55
Confidence in safety	6.33 ± 0.58	8.33 ± 0.58	7.33 ± 1.21
Overall score (pooled data)	6.73 ± 0.61	8.20 ± 0.56	7.47 ± 0.89

These subjective ratings should be interpreted cautiously, as they represent preliminary feedback from a small operator sample and do not constitute a definitive usability validation. They should be considered alongside the objective efficacy indicators reported in this paper.

#### Safety endpoint evaluation

In this study, the surgical safety of the novel interventional device was systematically evaluated from three dimensions: device integrity, embolus capture efficacy, and vascular histopathological changes. All indicators were assessed via intraoperative monitoring and immediate postoperative examination, with the results presented as follows.

##### (1) Device integrity

Immediate macroscopic examination was performed on the retrieved integrated system after the procedure. The results showed that the morphological structure of the device remained intact, with no abnormal conditions such as coating exfoliation, filament fracture, or structural damage observed. The integrity and tightness of the key functional components of the device were favorable, with no structural defects that would impair operational efficacy. Thrombus residue was visible inside the embolic protection filter.

##### (2) Embolus capture analysis

**Fig 6:**
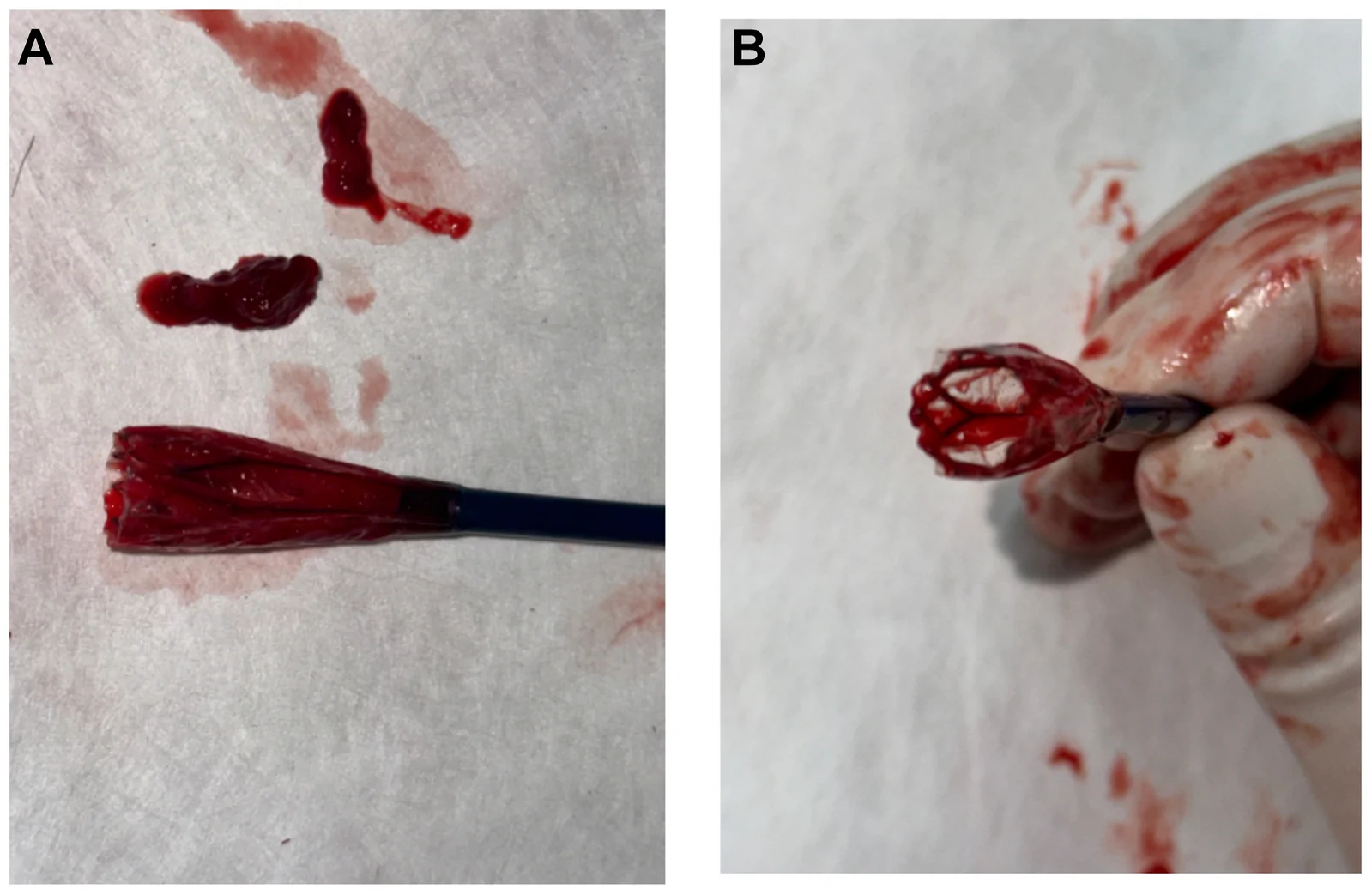
Macroscopic examination images of the integrity of the retrieved interventional device after the procedure. **A,** Thrombus debris flushed out from the embolic protection filter after the procedure; **B,** Captured thrombus residue was visible on the inner surface of the retrieved embolic protection filter, confirming that the system can effectively capture embolic debris generated during the intraoperative operation.

Gentle flushing of the embolic protection filter was performed after the procedure, and a small amount of intravascular thrombus was observed in the flushing fluid. This indicated that the embolic protection filter successfully captured part of the intravascular thrombus debris during the surgical operation, which preliminarily verified its in vivo embolic capture capability (Fig 6).

##### (3) Histopathological evaluation

**Fig 7:**
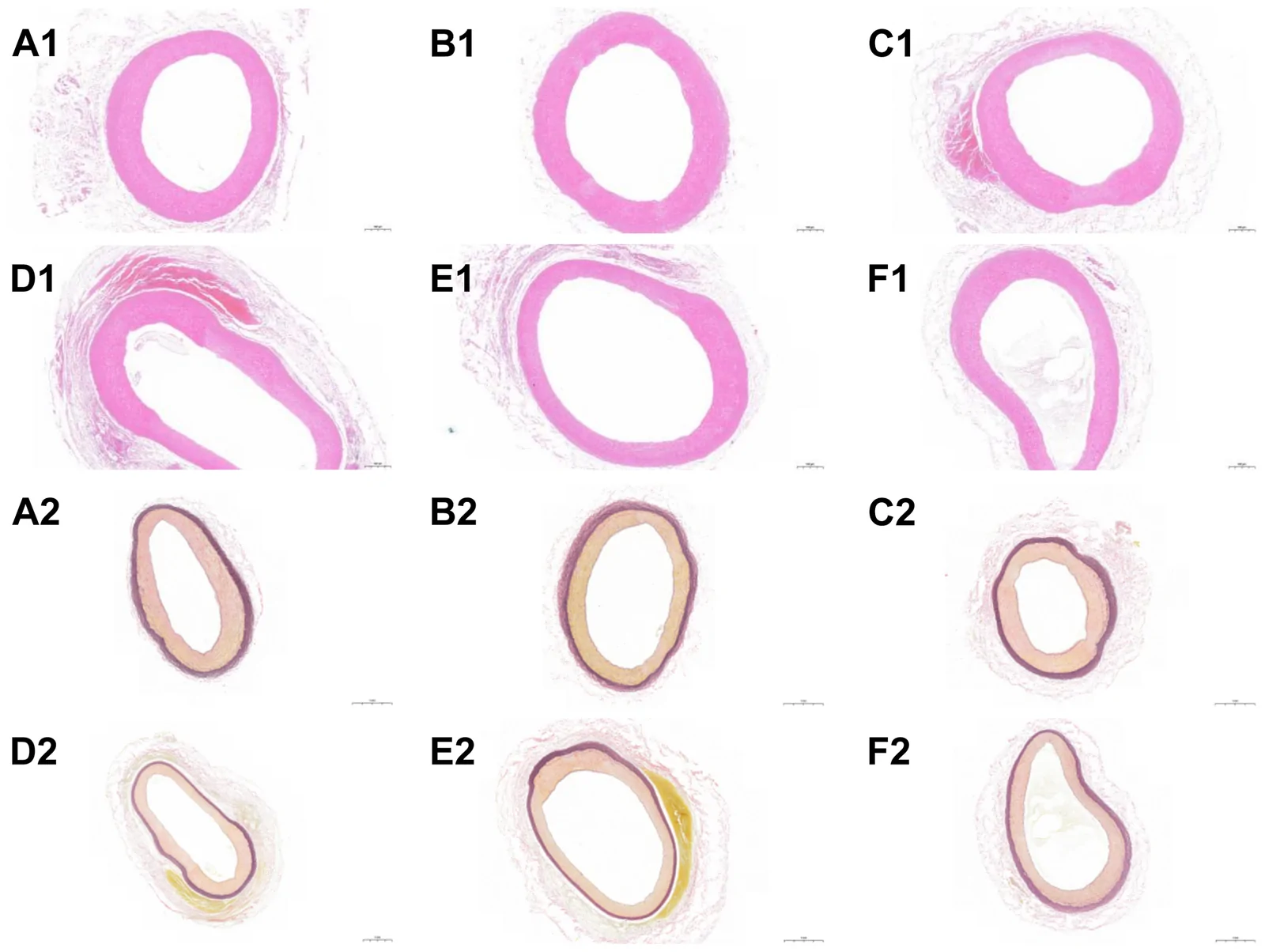
Results of hematoxylin and eosin (H&E) and elastic fiber staining of iliac artery segments under 4 × 10 magnification. **A1-F1,** H&E staining of the iliac arteries from the first to sixth experimental pigs, respectively, with mild vascular intimal injury observed in **(C1)** and **(F1)**; **A2-F2,** Elastic fiber staining of the iliac arteries from the first to sixth experimental pigs, respectively, with no obvious abnormalities observed in the elastic fiber staining.

Immediately after the procedure, iliac artery segments containing the puncture site were harvested. The specimens were fixed with 10% neutral buffered formalin, embedded in paraffin, and sectioned, followed by H&E staining and elastic fiber staining. Microscopic evaluation showed that the continuity of the vascular intima was well preserved, with only occasional focal mild injury, and no obvious laceration or defect was observed (Fig 7, *A1-F1*). The structure of the vascular elastic lamina was intact, with no manifestations of severe injury such as fracture or displacement. Only mild acute inflammatory cell infiltration was observed around the puncture site, without obvious edema, necrosis, or hematoma formation of the vascular wall. The overall morphological structure of the blood vessels was basically normal, and no severe vascular injury caused by device operation was found (Fig 7, *A2-F2*).

## Discussion

Thoracic aortic ISF is the core technique for supra-aortic branch reconstruction, whereas conventional strategies have long been plagued by three major clinical challenges: the risk of cerebral embolism, insufficient puncture accuracy, and the mutual exclusivity between cerebral protection and device access. This design may help address the aforementioned limitations. Preclinical experiments have verified that this system possesses both favorable safety and efficacy. Hereinafter, we conduct an in-depth discussion focusing on the core innovations of the system, and elucidate its technological breakthroughs and clinical value in the context of current research progress.

### Integrated design: resolving the access conflict of conventional strategies

In conventional ISF procedures, there is an inherent compatibility conflict in device access between the cerebral protection device and puncture operation: deployment of the embolic protection filter occupies the surgical access required for puncture and fenestration, rendering the puncture procedure unfeasible; whereas prioritizing the puncture access requires abandoning real-time cerebral protection, leaving patients exposed to the risk of cerebral embolism. This inherent limitation forces operators to face a persistent dilemma between “safety and operability”.

A key feature of this system is the integration of the embolic protection filter and the active steering directional puncture catheter into a single device, which enables simultaneous “whole-procedure cerebral protection” and “precision puncture” via a single access. This integrated design is not a simple superposition of devices, but a fundamental optimization of the surgical workflow: it eliminates the need for access switching and additional deployment of protection devices. In the in vivo experiments, all six experimental animals completed the full surgical workflow with a 100% success rate, no device-related complications occurred, and the total operative time was controlled at 38.92 ± 4.36 minutes. The score for “Degree of Procedural Simplification” in the operator feedback reached 7.50 ± 0.55 points. Compared with conventional strategies,^11^, ^12^, ^13^ these results demonstrate that this integrated design may simplify the surgical workflow, reduce operational complexity, and show promising feasibility in this preclinical setting.

### Active steering directional mechanism: Overcoming the challenge of puncture accuracy in complex anatomical scenarios

The anatomical complexity of the aortic arch (especially type Ⅱ and Ⅲ arches) is a key factor limiting the puncture success rate of ISF. Conventional puncture tools rely on the operator's hand feel and experience, making it difficult to achieve vertical puncture at the ostium of the target vessel. This tends to cause complications such as needle slippage and out-of-bounds puncture, which significantly increases surgical risk and operation time, and is one of the barriers restricting the popularization of ISF technology.^7^, ^8^, ^9^

In this study, the active steering directional mechanism allowed the puncture force line to be oriented approximately perpendicular to the stent graft by adjusting the force direction and angle of the catheter tip, thereby potentially reducing the degree of reliance on operator experience. The in vitro experimental results showed that this mechanism could achieve highly concentrated puncture sites within the projection area of the target vessel ostium across all types Ⅰ to Ⅲ arch scenarios, with a puncture coverage ratio of 41.9% to 68.8% and no out-of-bounds puncture observed in any case. Even under the type Ⅲ arch with the most restricted anatomical space, the effective puncture coverage ratio of the left subclavian artery and left common carotid artery remained at 41.9% and 44.0%, respectively, and the brachiocephalic trunk even showed an upward trend in puncture concentration. These findings suggesting that this mechanism has favorable adaptability to the most clinically complex anatomical scenarios.

Of note, the active steering directional design not only improves puncture accuracy, but also enables efficient fenestration. The mean fenestration time in the in vivo experiments was 0.15 ± 0.03 minutes (5-11 seconds), and this efficiency advantage is particularly prominent in direct comparison with existing fenestration devices. The currently recognized highly efficient Quick fenestrator still has a mean fenestration time of 0.67 ± 0.06 minutes (approximately 40 seconds), whereas the fenestration time of conventional laser and needle puncture is 5.50 ± 3.10 and 3.50 ± 1.50 minutes, respectively^11^ (Table VII). Of note, intraoperative observation found that after adjustment, the active steering directional puncture catheter also possessed the “fixation” and “position-limiting” functions as reported in the literature, which can stabilize the relative position between the catheter tip and the stent graft, reduce catheter displacement and force dispersion during puncture,^14^ and further improve the success rate of puncture and fenestration. Meanwhile, no clear linear association was observed between fenestration time and total procedure time in this study, which differs from the conclusion in existing studies that “puncture time is a key factor affecting total operative duration.”^11^^,^^15^ This preliminary finding indicates that this active steering directional mechanism can significantly reduce the time consumption and operational uncertainty of the puncture procedure, and is expected to alleviate the restriction of this step on the overall surgical efficiency. The highest score was observed in the dimension of directional accuracy in the operator feedback (7.83 ± 0.75 points), which is consistent with the favorable puncture performance observed in both the benchtop and in vivo experiments.

**Table VII:** Comparative performance of fenestration devices reported in the literature

Device type	Mean fenestration time, minutes	Integrated cerebral protection	References
Laser fenestration	5.50 ± 3.10	No	Wang et al,^11^ 2023
Needle fenestration	3.50 ± 1.50	No	Wang et al,^11^ 2023
Quick fenestrator	0.67 ± 0.06	No	Bai et al,^15^ 2021
Present integrated system	0.15 ± 0.03	Yes, 120 μm filter	Present study

Data for laser, needle, and Quick fenestrator are derived from published literature for contextual comparison only and do not represent head-to-head experimental testing.

### Targeted cerebral protection function: Addressing the core risk of high postoperative stroke incidence following ISF

The incidence of post-ISF stroke is as high as 6%,^5^^,^^6^ and its core inducement is cerebrovascular embolism caused by intraoperatively generated plaque debris, thrombus, and stent graft fragments larger than 100 μm.^16^^,^^17^ Studies have found that even patients with asymptomatic cerebral infarction present corresponding behavioral changes during long-term follow-up.^18^^,^^19^ Conventional strategies either lack whole procedure cerebral protection, or cannot perform puncture operations, whereas cerebral protection is implemented.

The cerebral protection function of the device in this study has clear targeting: the embolic protection filter adopts a mesh with a 120 μm pore size. In vitro experiments showed that the capture rates for microspheres of stroke-causing critical particle sizes of 120 and 150 μm reached 87.1% ± 2.7% and 98.1% ± 0.9%, respectively. This capture efficiency is comparable to the performance of clinically used cerebral protection devices reported in the literature,^20^^,^^21^ and may reduce the risk of symptomatic embolism. The result that the embolic protection filter successfully captured thrombus debris in the in vivo experiments verified its protective capability in a physiological blood flow environment. The 120 μm pore size reflects a balance between embolic protection and cerebral perfusion. Smaller pores could capture more sub-120 μm particles but may increase the risk of filter thrombosis and flow compromise, whereas larger pores may inadequately capture stroke-critical debris.^16^^,^^20^^,^^21^ In this study, capture efficiency was 87.1% for 120 μm but only 24.2% for 100 μm microspheres, indicating that smaller particles may still escape. The clinical significance of such microemboli remains uncertain, and future device refinements may address this residual risk. Notably, all in vitro capture experiments were performed using standardized spherical fluorescent microspheres, which differ in shape, surface texture, and mechanical properties from clinically relevant embolic materials such as irregular atherosclerotic plaque fragments and thrombus. Existing literature has consistently demonstrated that irregularly shaped debris is more easily captured by filter meshes than smooth spherical particles of the same size. Therefore, the capture rates reported in this study represent conservative estimates, and the actual embolic protection efficacy of the device in real clinical scenarios is likely to be higher.

The qualitative air bubble observation should be regarded strictly as hypothesis-generating; it provides no evidence of clinically meaningful air embolus protection. Intraoperative air introduction must continue to be meticulously avoided through standard flushing and deairing techniques.^22^^,^^23^

This design focuses on the intraoperative safety issues of ISF, and may help address the technical limitation of conventional strategies that protection and operation cannot be synchronized through the “whole procedure cerebral protection” mode. The in vitro experimental results and immediate in vivo observation findings have preliminarily confirmed its embolic capture capability, indicating its potential to provide embolism protection during ISF procedures, which lays a foundation for subsequent clinical validation.

## Study limitations and future perspectives

This study has several limitations.

The most critical limitation is the lack of a contemporaneous head-to-head control group. Literature-based comparisons (Table VII) are limited by inherent heterogeneity across studies and cannot substitute for rigorous controlled trials. All results presented are preliminary, and any claims regarding relative performance should be interpreted with extreme caution.

Next are some additional limitations.

First, the sample size was small (n = 6), and the in vivo experiments used an abdominal aortoiliac bifurcation model rather than a true thoracic aortic arch model. This model was selected to eliminate confounding anatomical complexity and enable standardized evaluation of device operability and immediate biosafety. However, differences in three-dimensional curvature, flow dynamics, and vessel compliance between this model, and the human thoracic arch may affect catheter maneuverability and filter stability in ways not fully captured herein; thus, the in vivo results should be interpreted as preliminary. Importantly, the in vitro experiments replicated human types Ⅰto Ⅲ aortic arch anatomies to validate core device performance—including puncture accuracy and embolic capture—under clinically realistic conditions.

Second, embolus capture efficiency was assessed using spherical fluorescent microspheres, which may not fully recapitulate the irregular morphology of atherosclerotic debris; consequently, the reported capture rates likely represent conservative estimates.

Third, the study employed healthy animals and assessed only immediate postoperative outcomes; therefore, no conclusions can be drawn regarding mid- or long-term safety, branch stent patency, or delayed device-related adverse events.

Fourth, the operator feedback assessment involved only two surgeons and is presented as preliminary observational data, not a definitive usability validation.

Future studies will focus on: (1) optimizing the puncture component design; (2) performing validation in atherosclerotic disease models with comprehensive neurological assessments and long-term follow-up; (3) conducting first-in-human clinical trials; (4) evaluating device usability in larger, blinded assessments involving operators with varying experience levels.

## Conclusions

In conclusion, the integrated cerebral protection and active steering directional puncture system developed in this study provides a potential solution to the core clinical dilemma of mutual exclusivity between intraoperative protection and procedural access in ISF, enabling both whole procedure cerebral protection and precision puncture. Preclinical experiments indicate that this system has promising efficacy and favorable immediate biosafety, offering a novel approach for supra-aortic branch reconstruction with potential for clinical translation.

## Author Contributions

Conception and design: XR, XL, JT, SJ, YT

Analysis and interpretation: XR, YT

Data collection: XR, XL, LZ, YZ, GA, DL, DA, LC, YD, HH

Writing the article: XR

Critical revision of the article: LZ, JT, SJ, YZ, GA, DL, DA, LC, YD, HH, YT

Final approval of the article: XR, XL, LZ, JT, SJ, YZ, GA, DL, DA, LC, YD, HH, YT

Statistical analysis: XR, YT

Obtained funding: YT

Overall responsibility: YT

Drs XR and XL contributed equally to this work and share co-first authorship.

## Declaration of generative AI and AI-assisted technologies in the writing process

No generative AI or AI-assisted technologies were used in the writing process of this manuscript.

## Ethics approval

All animal experimental protocols were approved by the Laboratory Animal Ethics Committee of Shanghai Key Laboratory of Vascular Surgery (Approval No. 2025-MS-LAT-D-62), and all procedures strictly complied with the 3R Principles for the Welfare and Use of Laboratory Animals and the Guide for the Care and Use of Laboratory Animals.

## Arrive guidelines

All in vivo animal experiments were performed and reported in strict accordance with the ARRIVE 2.0 guidelines for animal research reporting.

## Disclosures

The authors declare no conflicts of interest related to this study.
